# Efficiency of Lentiviral Vectors Pseudotyped with LCMV-G in Gene Transfer to Ldlr^−^^/−^ApoB^100/100^ Mice

**DOI:** 10.3390/genes17010060

**Published:** 2026-01-05

**Authors:** Alisa Nousiainen, Anna-Kaisa Ruotsalainen, Krista Hokkanen, Svetlana Laidinen, Ahmed Tawfek, Diana Schenkwein, Seppo Ylä-Herttuala

**Affiliations:** 1 A. I. Virtanen Institutefor Molecular Sciences, University of Eastern Finland, 70211 Kuopio, Finland; 2 Heart Center and Gene Therapy Unit, Kuopio University Hospital, 70210 Kuopio, Finland

**Keywords:** lentivirus vectors, gene therapy, pseudotyping

## Abstract

**Background/Objectives**: Lentiviral vectors (LVs) are most commonly pseudotyped with the vesicular stomatitis virus glycoprotein (VSV-G), which lends LVs a wide tropism as it uses the low-density lipoprotein receptor (LDLR) as the main receptor for cell entry. In some gene therapy and research applications, however, alternative pseudotypes can be useful. In this work, we characterized LVs pseudotyped with lymphocytic choriomeningitis virus (LCMV) glycoprotein, particularly in gene transfer to an LDLR-deficient mouse strain used to model cardiovascular disease, Ldlr^−/−^ApoB^100/100^. **Methods**: LCMV-LVs were used in vitro to test their transduction efficiency across a variety of cell types. In vivo, the gene transfer efficiency, LV-specific immune responses and biodistribution of VSV-G-LVs and LCMV-LVs were compared after systemic gene transfer. **Results**: In vitro, LCMV-LVs transduced all tested cell types at high efficiency without the use of transduction enhancers. In vivo, VSV-G-LVs showed a higher gene transfer efficiency at the same LV dose, but increasing the LCMV-LV dose enhanced the measured vector copy numbers. With both pseudotypes, most of the vector accrued in the liver, but with LCMV-LVs, a larger portion of the measured vector copies were found in the lungs. VSV-G-LVs also generated a higher titer of LV-specific IgG antibodies. The gene transfer efficiency of LCMV-LVs was affected by the mouse diet, with a high-fat diet decreasing the transduction. **Conclusions**: LCMV-LVs can be used as a substitute for VSV-G-LVs if an alternative pseudotype is required; however, they may require the use of a higher LV dose.

## 1. Introduction

Lentiviral vectors (LVs) are integrating vectors based on viruses from the *Lentivirus* genus of the *Retroviridae* family, most commonly those of the human immunodeficiency virus type 1 (HIV-1) [1]. Their ability to infect nondividing cells and integrate enables steady, long-term expression of the transgene and makes them useful in many applications in research and gene therapy. The most frequently used LVs today are third-generation, self-inactivating LVs, which use a split, four-plasmid production system and a deletion of the viral enhancer/promoter sequences from the vector’s long terminal repeats [2,3]. In a modification common to all LV generations, the HIV envelope protein has been removed from production and replaced with a separate pseudotyping construct, which encodes for the envelope surface protein that facilitates cell entry [2,4,5]; the standard envelope protein is the vesicular stomatitis virus glycoprotein (VSV-G).

It has been shown that VSV-G uses the low-density lipoprotein receptor (LDLR) and its family members for cell entry [6,7]. This conveys a wide tropism for LVs but may hinder transduction efficiency in cells and tissues where the target receptor is not expressed. This is the case with hematopoietic stem and progenitor cells, which express LDLR at very low levels; pseudotyping LVs with a baboon retroviral envelope glycoprotein (BaEV) instead of VSV-G has been shown to significantly increase LV transduction efficiency in these cells [8,9]. Several other alternative pseudotypes have been characterized: for example, the Cocal virus envelope reduces complement inactivation of LVs in comparison to VSV-G and has been suggested to be better suited for systemic delivery, while the Nipah virus pseudotype should better target ephrin B2 expressing cells and pass the liver sink [10,11]. However, the use of alternate pseudotypes can require significant adaptations to vector production and concentration protocols [12]. In this study, we chose to use the envelope protein of the lymphocytic choriomeningitis virus (LCMV) for LV pseudotyping.

LCMV is a noncytopathic enveloped virus from the Old World lineage of the *Mammarenavirus* genus in the *Arenaviridae* family. In LCMV, as other arenaviruses, the envelope glycoprotein precursor is encoded as a single transcript, which is cleaved by SKI-1 to form the stable signal peptide (SSP), and mature glycoproteins 1 and 2 (GP1 and GP2). The cleaved SSP, GP1, and GP2 form a three-part complex on the virus surface: GP1 binds to the receptor, following which the virus enters the cell through receptor-mediated endocytosis. In the late endosome, the GP1 detaches from the complex due to a conformation change triggered by the low pH, and GP2 enables fusion of the membranes of the virus and the endosome [13]. The main cellular receptor for LCMV is dystroglycan, but it can also use C-type lectins for cell entry [14,15]. Previous studies on vectors pseudotyped with LCMV-glycoprotein have shown that LCMV-LVs can be produced and concentrated with the same protocols as VSV-G-LVs with less toxicity in the producer cells and with equivalent infectious titers [16], with the use of the WE-strain glycoprotein leading to higher titers than the use of the Arm53b strain glycoprotein [17]. In vivo, LCMV-LVs were able to correct the bleeding phenotype in a mouse model for hemophilia while incurring minimal systemic and hepatic toxicity [18].

In this work, we have characterized LVs pseudotyped with the LCMV WE-strain glycoprotein in vitro and in gene transfer to the Ldlr^−/−^ApoB^100/100^ (Ldlr-KO) mouse strain [19], which is used to model hyperlipidemia and atherosclerosis, and which we have previously used in adenoviral gene therapy studies [20,21]. The hyperlipidemic Ldlr-KO mice represent an opportunity where an alternative for VSV-G might be used, as LDLR, the main entry receptor of VSV-G, is absent. It is also worthwhile to characterize the performance of pseudotypes across different mouse strains, as the strain and even the diet of the mice can affect gene transfer outcomes [21,22]. LCMV-LVs were found to efficiently transduce a variety of cell types in vitro without reduction in efficiency from LDL-supplementation of the transduction media. In vivo analysis showed that Ldlr-KO mice and C57BL/6J mice were transduced at a similar efficiency, but a high-fat diet (HFD) in Ldlr-KO mice reduced the gene transfer efficacy. A biodistribution analysis showed that, as with VSV-G-LVs, LCMV-LVs accumulate mostly in the liver. Despite the lack of functional LDLR in the mouse model, VSV-G-LVs were more efficient in in vivo gene transfer at the same vector particle (VP) dose after systemic delivery to Ldlr-KO mice; however, increasing the LCMV-LV dose increased the gene transfer efficiency, suggesting that LCMV-LVs can be used if an alternative pseudotype to VSV-G is required.

## 2. Materials and Methods

### 2.1. Lentivirus Vector Production

**Figure 1:**
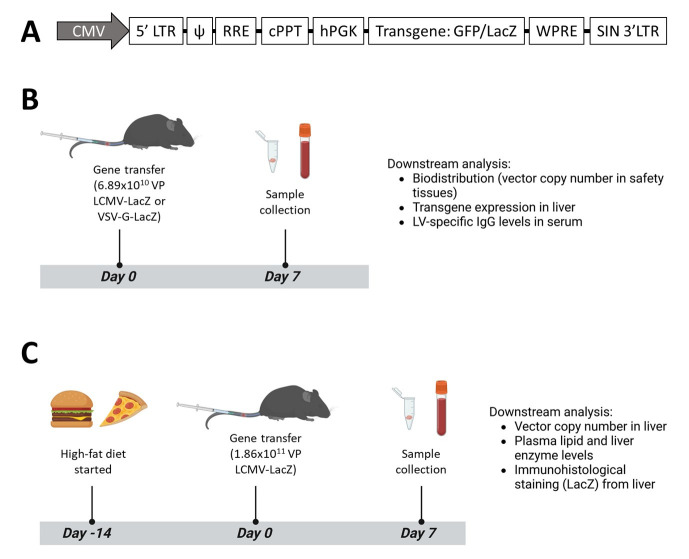
(**A**) A schematic representation of the LV transgene constructs used in this study. (**B**) In vivo experiment design of initial pseudotype comparison experiment. (**C**) The experiment design to assess the effect of mouse diet on LCMV-LV gene transfer efficiency (HFD started only for the HFD group). Panels (**B**) and (**C**) created with BioRender.

All LV modifications used in the study are listed in Table 1. The pLV-hPGK-LacZ transgene construct sequence was created by back-translation of the *Escherichia coli* beta-galactosidase protein (Uniprot: Q8VNN2) with EMBOSS Backtranseq; the sequence was synthesized and then cloned to the pLV-backbone. The LCMV-pseudotyping construct was generated by synthesis of the coding sequence for the lymphocytic choriomeningitis virus glycoprotein C from the WE-strain (GenBank: AJ297484). Two ApoI-sites were deleted from the sequence by single-nucleotide substitutions (nucleotides 456 and 1053, T > C, no aa change), and the sequence was synthesized and cloned to the pCMV-VSV-G plasmid to replace the VSV-G-gene. Transgene synthesis and plasmid cloning was conducted via Genewiz (Leipzig, Germany). For EGFP-expressing LVs, a pLV-hPGK-GFP-WPRE transgene construct plasmid was used in LV production; a schematic representation of the transgene constructs used in this study is presented in Figure 1A. The third-generation LVs used in the study were produced in the National Virus Vector Laboratory of Biocenter Kuopio, Finland, by calcium phosphate transfection of 293T cells and concentrated by ultracentrifugation [23]. Briefly, the producer cells were transfected in 525 cm^2^ triple-layer flasks with the LV production plasmids (transgene plasmid pLV, pseudotyping plasmid pCMV-VSV-G or pCMV-LCMV, and packaging plasmids pMDLg-pRRE and pRSV-Rev, ratio of 2.4:1:1.04:1.11, respectively; see Supplementary File S1 for plasmid maps). Ultracentrifugation was performed at 45,952 rcf (19,500 rpm, Optima L-80 XP Ultracentrifuge, Beckman Coulter, Brea, CA, USA) for 130 min at 18 °C. VP titration was performed with the Alliance HIV-1 p24 Antigen ELISA Kit (NEK050, PerkinElmer, Waltham, MA, USA).

### 2.2. In Vitro Testing of LCMV-Pseudotyped LVs

LV transduction efficiency was tested in HEK293 (human embryonic kidney cells, ATCC CRL-1573, Manassas, VA, USA), MRC-5 (human embryonic lung fibroblasts, ATCC CCL-171), AML-12 (mouse hepatocytes, ATCC CRL-2254), and Huh7 (human hepatocellular carcinoma, Cytion CLS Cell Line Service #300156, Heidelberg, Germany) cell lines. Cells were plated at 1 × 10^5^ cells/well the day before transduction and transduced with EGFP-expressing LVs pseudotyped with LCMV-G in a dilution series of 1:10,000–1:1,000,000 in a total volume of 1 mL. Cell number at the time of transduction was counted from control wells of each cell line. The transduction incubation lasted 24 h, after which the transduction medium was replaced with normal culture medium. EGFP-expression was analyzed by flow cytometry (CytoFLEX S, Beckman Coulter, Brea, CA, USA) 4 days post-transduction to measure the percentage of EGFP-expressing cells and calculate the infectious titer of the vector (TU/mL). Testing of low-density lipoprotein (LDL)-inhibition of transduction efficiency was performed on 293T cells: transduction medium was supplemented with LDL (I34360B, Thermo Scientific, Waltham, MA, USA) at a concentration of 2 mmol/L, and the cells were transduced with LCMV-LVs or VSV-G-LVs at 1 × 10^4^ vector particles/cell as measured by the p24 particle titer. For control, cells were similarly transduced in regular culture medium without LDL supplementation. The transduction incubation lasted 4 h, after which the transduction medium was replaced with normal culture medium. Vector copy number (VCN) in transduced cells was analyzed 6 days post-transduction with droplet digital PCR (ddPCR).

### 2.3. In Vivo Gene Transfer

Gene transfers were performed on Ldlr^−/−^ApoB^100/100^ mice in C57BL/6J background (strain 003000, The Jackson Laboratory, Bar Harbor, ME, USA; C57BL/6JOlaHsd mice used to refresh the population in-house) or C57BL/6JOlaHsd (Envigo) using the highest available dose of LV. The mice diets were the standard chow diet (Teklad Global 16% Protein Rodent Diet, 2016S, Envigo, Indianapolis, IN, USA) or, where mentioned, a HFD (TD.88137, Envigo) containing 42% of energy from fat. The HFD was started 2 weeks pre-gene transfer and continued until study endpoint. See Figure 1 for in vivo experiment design. For the initial pseudotype test comparison, 6.89 × 10^10^ VP/mouse of VSV-G-LacZ or LCMV-LacZ was administered intravenously (i.v) through the tail vein to 7-month-old male mice. The treatments were administered to littermate controls and gene transfer operations were performed by a researcher blinded to the treatment groups. For the higher-dose LCMV-pseudotype test, 1.86 × 10^11^ VP was administered i.v. to 3-month-old female Ldlr^−/−^ApoB^100/100^ mice and 8-month-old female C57BL/6JOlaHsd mice. Food and water were provided ad libitum, and the mice were sacrificed 7 days post-gene transfer. For a “no vector” control of plasma liver enzyme levels, blood samples were collected from female 4-month-old Ldlr^−/−^ApoB^100/100^ mice after 1 week of HFD. The mice were kept in standard housing conditions in the University of Eastern Finland Laboratory Animal Centre, Kuopio, Finland. All animal experiments and procedures used in this study were approved by the Finnish National Animal Experiment Board under the licenses ESAVI-2021-014193 and ESAVI-2024-011569 and carried out in accordance with animal welfare legislation.

### 2.4. Histological Staining and Analysis of Mouse Tissue Samples

The mice were euthanized by CO_2_, and tissues were collected for histological analysis. Tissues were fixed overnight in 4% PFA in DPBS then stored in DPBS until tissue processing and paraffin-block embedding of tissue samples. Immunohistochemistry staining of bacterial β-galactosidase expression following LCMV-LacZ gene transfer was undertaken from 5 µm tissue sections using a β-galactosidase polyclonal antibody (Invitrogen, Carlsbad, CA, USA, A-11132) at a dilution of 1:1000 and biotinylated goat anti-rabbit IgG antibody (Vector laboratories, Burlingame, CA, USA, BA-1000) at a dilution of 1:200. Staining was undertaken using the Vectastain ABC-HRP kit (Vector laboratories, Burlingame, CA, USA, PK-6100) and DAB substrate kit (Vector laboratories, Burlingame, CA, USA, SK-4100); sections were counterstained with Harris hematoxylin. All histological imaging was conducted with an ECLIPSE Ni-E microscope (Nikon Instruments, Tokyo, Japan). Chromogen liver staining was analyzed with ImageJ (Fiji, version 2.3.0) by quantifying the percentage of LacZ-positive area from representative liver images. The immunohistological staining and analysis was performed by a researcher blinded to the sample groups.

### 2.5. Analysis of Mouse Serum and Plasma Samples

Heparin plasma lipid levels and liver enzymes (7 days post-gene transfer) were measured by Movet animal diagnostic laboratory, Kuopio, Finland. LV-specific antibodies were analyzed from mouse serum collected 7 days post-gene transfer: a Nunc Medisorp (Thermo Scientific, 446470) plate was coated overnight with LV (100 ng of p24 per well; the wells were coated with the LV used in the gene transfer, so LCMV-LacZ-treated mouse serum was analyzed in wells coated with LCMV-LacZ, and VSV-G-LacZ-treated mouse serum in wells coated with VSV-G-LacZ). The plate was washed with washing buffer (DPBS + 0.05% Tween-20) and then blocked with 300 µL/well of DBPS + 0.5% BSA + 0.05% Tween-20 for 2 h at 37 °C. Serum samples were diluted to 1:100, 1:200, 1:500, and 1:1000; after removal of the blocking buffer, 200 µL/well of the diluted sample was incubated at 37 °C for 90 min. The HIV-1 p24 antibody (Invitrogen, MA17381) was used as a positive control. The plate was washed 3 times with the washing buffer, then the secondary antibody (1:1000 dilution of Goat anti-mouse IgG, peroxidase conjugated, Thermo Scientific, 32430) was incubated at 37 °C for 1 h. The plate was washed once, and the substrate (1-Step Ultra TMB-ELISA, Thermo Scientific, 34028) was added and incubated for 20 min at RT. The reaction was stopped with 2 M H_2_SO_4_, and absorbance at 450 nm was measured with the ClarioSTAR plate reader.

### 2.6. Droplet Digital PCR Analysis of Vector Copy Number and LV Biodistribution

Mouse tissues collected for nucleotide analysis were flash-frozen in liquid nitrogen and stored at −70 °C until nucleic acid extraction. DNA was extracted with the DNeasy Blood & Tissue-kit (Qiagen, Hilden, Germany, 69506) for VCN measurement and biodistribution analysis from the liver, spleen, kidney, heart, testis, and lung samples. TRIreagent (Invitrogen, AM9738) was used for RNA extraction from the liver at 7 days post-gene transfer. RNA samples were treated with DNase (TURBO DNase, Invitrogen 2238), and the DNase-treated RNA was then used to generate cDNA (RevertAid reverse transcriptase, ThermoScientific EP0442, and Random hexamer primer, ThermoScientific S0142), Bio-Rad’s ddPCR system was used to quantitate VCN from DNA samples using a WPRE-assay (forward primer: CACTGACAATTCCGTGGTGT; reverse primer: CAGAATCCAGGTGGCAACA; FAM probe: ACGTCCTTTCCATGGCTGCTCGCCT) or a LacZ assay (forward: GTACACCCCCTACGTGTTC; reverse primer: GTTGAACTGGAAGTCGCC; FAM probe: ACGGCCTGAGGTGCGGCACC) and an Rpp30 mouse genome reference assay (Bio-Rad, Hercules, CA, USA, dMmuCNS822293939).

### 2.7. Statistics

Statistical analysis was conducted with the GraphPad Prism 5 for Windows, version 5.03, GraphPad Software (Boston, MA, USA). Statistical tests are specified in the figure legends where the analyzed data is presented. To ensure that the appropriate statistical analysis was performed, equal variances were confirmed with Bartlett’s test.

Power calculations for the in vivo study groups were performed with the formula $n=\frac{2\;{SD}^{2}{\;(Z^{a/2}\;+\;Z^{\beta })}^{2}}{d^{2}}$, as in [24], considering the vector transgene copy number the main variable, with standard deviation of 0.5, and effect size of 1, 80% power, and 5% type 1 error.

## 3. Results

### 3.1. In Vitro Characterization of LCMV-Pseudotyped LVs

**Figure 2:**
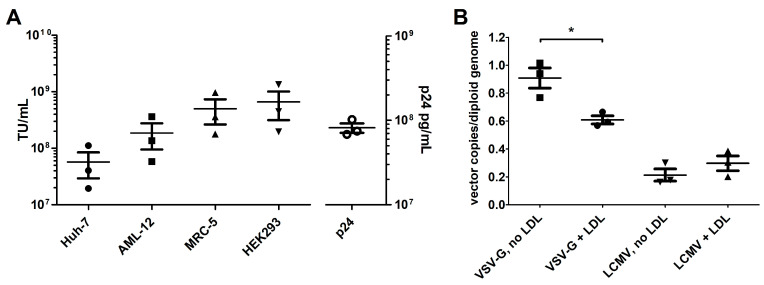
(**A**) The infectious titer (TU/mL) of LCMV-GFP, as measured in Huh-7, AML-12, MRC-5, and HEK293 cells, and the capsid protein p24 titer (pg/mL, right *y*-axis), as measured by ELISA. N = 3 different LV production lots; lines display mean and SEM. (**B**) The LV copy number value measured from 293T cells after transduction with VSV-G-pseudotyped or LCMV-pseudotyped LV in the presence of 2 mmol/L LDL supplementation in the transduction medium (+LDL) or without it (no LDL). N = 3, lines show mean and standard error of the mean (SEM). Statistical analysis: one-way ANOVA and Tukey’s multiple comparison test, * *p* < 0.05.

The LCMV-pseudotype was first tested in vitro to assess its transduction efficiency across different cell types. A functional titration was conducted using three separate production lots of LCMV-LVs encoding GFP. LCMV-LVs efficiently transduced all tested cells; human embryonic kidney cell-derived HEK293 and human embryonic lung fibroblast-derived MRC-5 cells had the highest measured functional titer (Figure 2A). The tested liver-derived cell lines, human hepatocellular carcinoma-derived Huh-7, and mouse hepatocyte-derived AML12 were also efficiently transduced, with all measured titers exceeding 1 × 10^7^ TU/mL without the use of transduction enhancers. For the titers measured in our standard vector production of VSV-G-LVs using HeLa cells, see Supplementary Figure S1.

The transduction efficiency of LCMV-LVs and VSV-G- LVs was also tested on 293T cells with 2 mmol/L LDL supplementation. The transduction efficiency of VSV-G-LVs was reduced in the presence of LDL (Figure 2B), but the VCN of integrated LCMV-LVs was not affected. The vector dose chosen for the LDL supplementation test was 1 × 10^4^ vector particles, as measured by the capsid protein p24 titer, and as such, the TU used was different between the pseudotypes. This is reflected in the measured VCN, as the transduction with VSV-G-LVs and no LDL-supplementation led to an approximately 4-fold higher copy number than the comparable LCMV-LV transduction.

### 3.2. LCMV-LV-Mediated Gene Transfer to Ldlr-KO Mouse Liver Is Lower than with VSV-G-LVs

**Figure 3:**
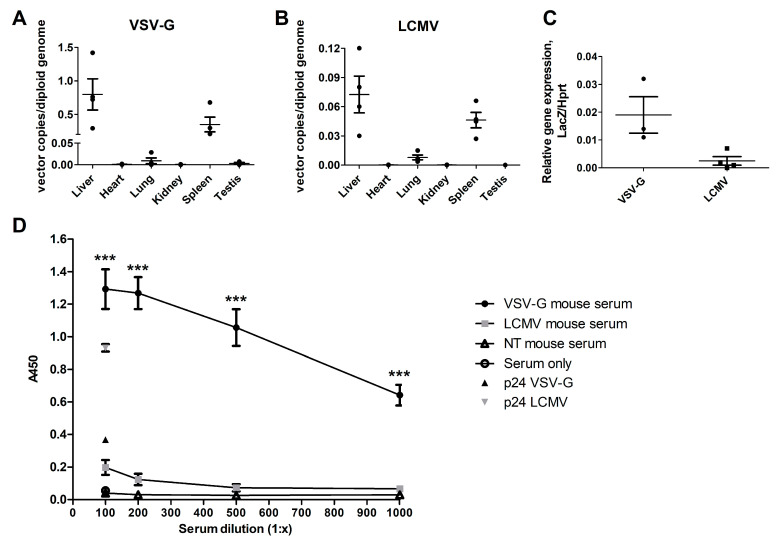
(**A**) Biodistribution of VSV-G-pseudotyped LV measured as vector copy number value per diploid genome analyzed with ddPCR from the collected tissues 7 days post intravenous gene transfer through the tail vein in Ldlr^−/−^ApoB^100/100^ mice. N = 4; each dot represents an individual mouse. Lines show mean and SEM. (**B**) Biodistribution of LCMV-pseudotyped LV measured as vector copies per diploid genome analyzed with ddPCR from the collected tissues 7 days post intravenous gene transfer through the tail vein in Ldlr^−/−^ApoB^100/100^ mice. N = 4; each dot represents an individual mouse. Lines show mean and SEM. (**C**) A comparison of the transgene (LacZ) expression measured from liver RNA following gene transfer with VSV-G- or LCMV-pseudotyped LV-LacZ, normalized to Hprt expression (same experiment as in A and B, measured with ddPCR). (**D**) Analysis of LV-specific IgG-antibodies with ELISA from mouse sera 7 days post-gene transfer with VSV-G or LCMV pseudotyped LVs: anti-p24 antibody on LCMV-LV-coated wells (p24 LCMV), anti-p24 antibody on VSV-G-LV-coated wells (p24 VSV-G), non-treated (NT) mice sera, and non-LV coated (serum only) wells as controls. N = 4 for treated mice and n = 2 for controls, mean and SEM. Statistical analysis: two-way ANOVA and Bonferroni posttest, *** *p* < 0.001.

The in vivo gene transfer efficiency of LCMV- and VSV-G-pseudotyped LVs was compared in a systemic gene transfer to the Ldlr-KO mouse model. Seven days post-gene transfer, the mice were sacrificed via tail vein injection, and the VCN and transgene expression were analyzed from the liver, heart, lung, kidney, spleen, and testis. In both groups, the highest VCN was measured from the liver (Figure 3A,B). With the same dose of 6.89 × 10^10^ VP/mouse, VSV-G-LVs incurred approximately 10-fold higher hepatic VCN and similarly higher transgene expression than LCMV-LVs (Figure 3A–C; VSV-G VCN 0.8 ± 0.23 copies per diploid genome and LCMV VCN 0.07 ± 0.01 copies per diploid genome (mean ± SEM)). After the liver, both VSV-G-LV and LCMV-LV most efficiently transduced the spleen, followed by the lung; in all other tissues, the measured VCN was so low as to be negligible. The VCN measured from the lung samples of the LCMV-LV group (0.008) was similar to that measured from the VSV-G-LV group (0.009) despite the considerable difference in the liver vector copy number, indicating that in the LCMV-LVs, a higher proportion of the vector accrued in the lungs.

To assess the immune response to the gene transfer, the IgG antibody response to LCMV- and VSV-G-LVs was measured from the mouse serum collected at the same time point. The mice treated with VSV-G-LV developed significantly higher titers of LV-specific antibodies than the mice treated with the same particle amount of LCMV-LV (Figure 3D).

### 3.3. HFD Reduces LCMV-LV-Mediated Gene Transfer to Ldlr-KO Mouse Liver

**Figure 4:**
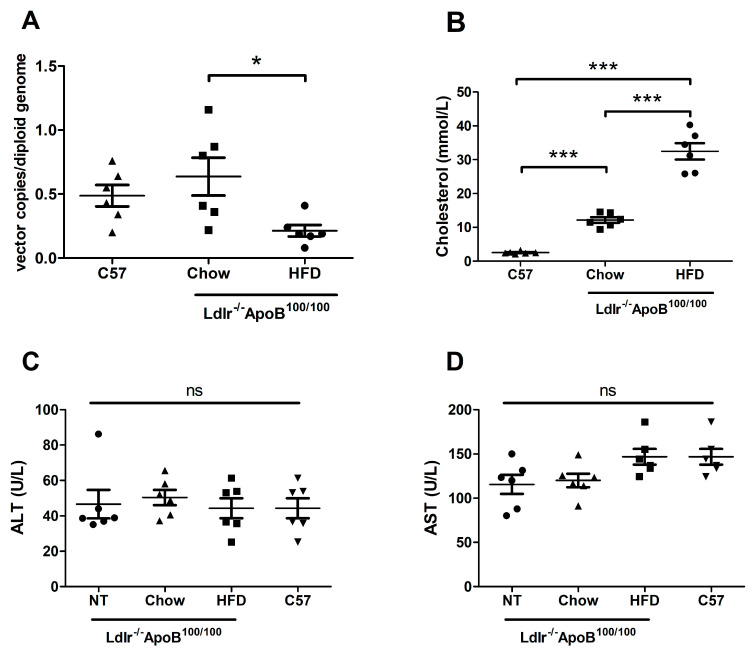
(**A**) The LV copy number per diploid genome measured from the liver 7 days post intravenous gene transfer through the tail vein with LCMV-LV in Ldlr^−/−^ApoB^100/100^ mice on a high-fat diet (HFD) or a chow diet or the C57BL/6JOlaHsd (C57) control mice. (**B**) The total cholesterol (mmol/L) in plasma measured 7 days post-gene transfer from the mice in (**A**). (**C**) The liver enzyme ALT (U/L) in plasma measured from the mice in A and non-treated (NT) control Ldlr^−/−^ApoB^100/100^ mice after 1 week of HFD. (**D**) The liver enzyme AST (U/L) in plasma measured from the mice in C. In all panels, n = 6, and each dot represents an individual mouse; lines show mean and SEM; statistical analysis: one-way ANOVA and Tukey’s multiple comparison test, where ns = no significance; * *p* < 0.05, *** *p* < 0.001.

**Figure 5:**
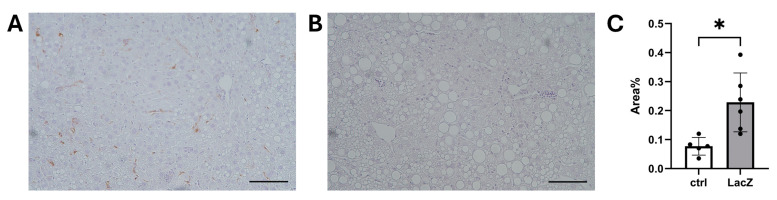
Immunostaining of bacterial β-galactosidase expression from mouse liver samples 7 days post-gene transfer (i.v. injection of LCMV-LacZ through the tail vein). Representative images at 20× magnification of (**A**) LCMV-LacZ-treated mice vs. (**B**) controls; scale bars: 100 μm. (**C**) Analysis of stained area, presented as a percentage of the LacZ-positive area of the whole image. Each data point represents the percentage measured from an individual mouse; lines show mean and SEM. Statistical analysis: Unpaired two-tailed *t*-test, * *p* < 0.05.

The effect of the mouse diet on LCMV-LV gene transfer efficiency was assessed by intravenous injection of 1.86 × 10^11^ VP/mouse. A HFD started two weeks prior to gene transfer was enough to significantly reduce the VCN measured from the liver 7 days post-gene transfer in comparison to the regular chow diet (Figure 4A) in the Ldlr-KO mice. However, the Ldlr-KO mice on the chow diet had a comparable liver VCN to control C57BL/6J mice on the same diet, despite significantly higher blood cholesterol levels (Figure 4B), indicating that higher blood lipid levels were not the cause of the HFD-related decrease. Analysis of liver enzymes alanine aminotransferase (ALT; Figure 4C) and aspartate aminotransferase (AST; Figure 4D) in mouse plasma showed no statistically significant difference between the LCMV-LV treated groups or in comparison to a control group that received no LV treatment. The mean liver VCN of the chow-fed Ldlr-KO mice was 0.64 copies per diploid genome; analysis by IHC staining of the transgene from the liver showed that this corresponded to 0.2% stained area (Figure 5).

## 4. Discussion

In this work, we set out to characterize the LCMV-LVs as a possible alternative for the standard VSV-G-LVs, especially in Ldlr-KO mice. As reported previously, in our hands, high-titer LCMV-LVs could be produced with no alterations to the standard VSV-G-LV production protocol. This makes a notable difference to, e.g., the BaEV-LVs, which have been shown to cause significant syncytia formation in producer cells, and which necessitates changes to the vector production [12,25]. In vitro, LCMV-LVs could efficiently transduce a variety of cell types of both human and mouse origin. As the main receptor for LCMV is the ubiquitously expressed dystroglycan [14], a wide tropism is expected. From a biosafety aspect, a tropism limited to the target cell type would be ideal to minimize gene therapy side effects; however, a wider tropism broadens the general usability of the pseudotype. From a translational viewpoint, cross-species transduction capabilities are ideal as preclinical testing performed in vivo in a mouse model should be as closely applicable to human patients as possible. The seroprevalence of LCMV antibodies, which could neutralize the pseudotype, has been cited as low (<10%) in analyzed human populations [26,27], which further broadens the potential use of this pseudotype. Both in vitro and in vivo, it was also determined that an increase in LDL concentration at time of transduction did not impede transduction efficiency, suggesting that LCMV-LVs can be used in transduction setups requiring high cholesterol conditions.

In a comparison of LCMV-LVs and VSV-G-LVs in systemic in vivo gene transfer to Ldlr-KO mice, we saw that the same VP dose led to a 10-fold higher gene transfer efficiency in VSV-G-LVs compared to LCMV-LVs. VSV-G-LVs, despite their affinity for the LDLR, are capable of efficient gene transfer in adult Ldlr-KO mice. However, this is well in line with the finding that LDLR family members, such as low-density lipoprotein receptor-related protein 1 (LRP1), act as alternative receptors of VSV-G [6,7]. *Lrp1*, like *Ldlr*, is highly expressed in the liver [28,29], and in the VSV-G-LV biodistribution analysis, the liver was the most highly transduced tissue. All in all, the biodistribution analysis of VSV-G-LVs in the Ldlr-KO mouse strain corresponds to the previously published biodistribution studies conducted in normal BALB/c mice [30]. The LCMV-LV, in comparison to VSV-G-LV and in proportion to the overall gene transfer efficiency, transduced the lungs more effectively, suggesting that the pseudotype could potentially be used in gene transfer aimed at the lungs.

The follow-up time of 7 days post-gene transfer is not long enough for the proper assessment of a transgene-specific adaptive immune response, which has been shown to peak two weeks post-LV gene transfer [31]. We therefore only measured the LV-specific IgG-antibodies in mouse serum following gene transfer with VSV-G- or LCMV-LVs. The antibody titers generated by VSV-G-LVs were significantly higher than those generated by LCMV-LVs. This reflects the gene transfer efficiency, as the VSV-G-LV generated a higher level of transgene copies in mouse tissues; however, it also indicates that there was likely not a high amount of immunogenic LCMV-LV transduction in a tissue type not covered by our biodistribution analysis of selected safety tissues. Ideally, we would have been able to additionally confirm the results with LCMV- and VSV-specific positive control serums; however, the p24 antibody control used indicated efficient coating of wells in both vector groups. There is no reason to suspect that the antibody response generated by the LVs in mice would be restricted only to the glycoprotein used in pseudotyping, rather than, for example, also the p24 capsid protein, which is a distinct antigen and routinely used in human HIV diagnosis [32]. As the transduction of antigen-presenting cells is considered one of the key factors in the induction of an adaptive immune response [33], the difference in gene transfer efficiency can explain the difference in antibody titers. Furthermore, antibody responses against LCMV have been shown to develop late in the infection cycle and at low titers [34,35,36,37], unlike responses against VSV [38], and it may be that the difference in antibody titers would have been less significant if analyzed at a later time point.

It has previously been reported that LV-mediated gene transfer leads to a swift innate immune response, independent of the pseudotype, and that this response reduces the VCN in the liver within the first 24 h after gene transfer [39]. In this study, we did not try to mitigate this response by immune suppression as we wanted to be able to compare the adaptive immune responses to the tested pseudotypes; however, especially in gene therapy applications, prophylactic immune suppression could increase the gene transfer efficiency and lower the LV dose required for the generation of a therapeutic effect.

In vivo gene transfer is sensitive to variables and depends not only on the mouse strain used but can also be affected by the mouse diet [21,22]. In this work, we tested whether LCMV-LV gene transfer was affected by mouse diet in the Ldlr-KO strain. We also tested whether gene transfer efficiency in the Ldlr-KO strain differed from that in the background C57BL/6J strain. HFD is commonly used to accelerate the development of atherosclerosis in mice [40], and HFD-generated effects on gene transfer can affect the usefulness of the pseudotype in studies of cardiovascular disease as well as hyperlipidemia. A HFD started two weeks pre-gene transfer was enough to significantly lower gene transfer to the liver in the Ldlr-KO strain; however, there was no significant difference between the Ldlr-KO mice and the normal mice on the standard chow diet, despite a 5-fold difference in total cholesterol levels. This suggests that although the HFD significantly increases the plasma lipid levels, the effect on gene transfer efficiency is not conveyed through that.

In this study, as the focus was on LV pseudotypes, phagocytosis shielding or transgene construct enhancements such as microRNA targets that silence the transgene in non-target tissues were not used. These vector modifications have been shown to improve gene transfer efficiency as well as transgene longevity [41,42]. In long-term studies, and especially in gene therapy applications, the use of modifications such as these would be beneficial. In this study, the ratio of detected transgene expression to measured VCN was quite low; while this can partly be explained by the lack of vector optimization, transgene expression following LV gene transfer can take two weeks post-gene transfer for peak expression [31].

While the LCMV-pseudotype is not a direct improvement on the standard VSV-G-pseudotype in the models tested here, it can be produced with the same small-scale production protocol and used to transduce both human and mouse cells in vitro efficiently without transduction enhancers. Furthermore, unlike VSV-G, it is not impeded by LDL-supplementation; any applications where a VSV-G substitute or comparison is required, especially in hyperlipidemic settings, may make use of LCMV-LVs. Furthermore, in vivo applications targeting the lungs may benefit from the use of LCMV-LVs, but confirmation of this will require further studies with longer time points.

## Figures and Tables

**Table 1:** LV transgenes and pseudotypes used in the study.

	Type	Function
Pseudotyping constructs	VSV-G	Control envelope
	LCMV	Pseudotype tested for characterization
Transgene constructs	hPGK-EGFP	Reporter for in vitro testing
	hPGK-LacZ	Reporter for in vivo gene transfer

## Data Availability

The original contributions presented in this study are included in the article/Supplementary Materials. Further inquiries can be directed to the corresponding author.

## Supplementary Materials

The following supporting information can be downloaded at: https://www.mdpi.com/article/10.3390/genes17010060/s1, Figure S1: Capsid protein (p24 pg/mL) and infectious titers (TU/mL) of VSV-G-GFP LV production lots; p24 measured by ELISA and TU/mL measured in HeLa cells (ATCC CCL-2); Supplementary File S1: LV production plasmid maps.

## Abbreviations

The following abbreviations are used in this manuscript:

LV	Lentiviral vector
VSV-G	Vesicular stomatitis virus glycoprotein
LDLR	Low-density lipoprotein receptor
LCMV	Lymphocytic choriomeningitis virus
HIV-1	Human immunodeficiency virus type 1
BaEV	Baboon retroviral envelope glycoprotein
SSP	Stable signal peptide
GP1	Glycoprotein 1
GP2	Glycoprotein 2
Ldlr-KO	Ldlr^−/−^ApoB^100/100^
HFD	High-fat diet
VP	Vector particle
ddPCR	Droplet digital PCR
i.v.	Intravenous
VCN	Vector copy number
SEM	Standard error of the mean
ALT	Alanine aminotransferase
AST	Aspartate aminotransferase
LDL	Low-density lipoprotein
TU/mL	LV infectious titer, transducing units per mL
LRP1	low-density lipoprotein receptor-related protein 1
