# Supplementary material for: Efficiency of Lentiviral Vectors Pseudotyped with LCMV-G in Gene Transfer to Ldlr−/−ApoB100/100 Mice
Source: Genes (Basel). 2026 Jan 5;17(1):60. doi: 10.3390/genes17010060 (PMC12841177; doi:10.3390/genes17010060)
Supplement: Supplementary file 1 [file genes-17-00060-s001.zip › Supplementary Figure S1.pdf]

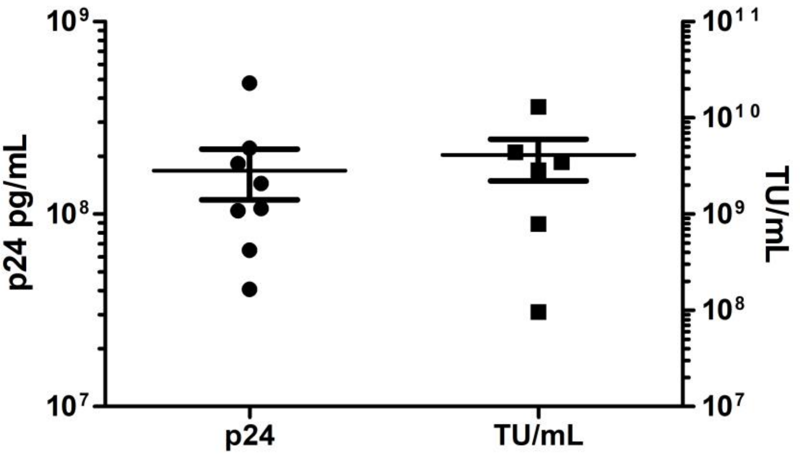

Supplementary Figure S1. Capsid protein (p24 pg/mL) and infectious titers (TU/mL) of VSV-G-GFP LV production lots; p24 measured by ELISA and TU/mL measured in HeLa cells (ATCC CCL-2). Each dot represents one LV lot, lines show mean and standard error of the mean.
