# Supplementary figures and images for: Efficiency of Lentiviral Vectors Pseudotyped with LCMV-G in Gene Transfer to Ldlr−/−ApoB100/100 Mice
Source: Genes (Basel). 2026 Jan 5;17(1):60. doi: 10.3390/genes17010060 (PMC12841177; doi:10.3390/genes17010060)

## Supplementary File S1. LV production plasmid maps

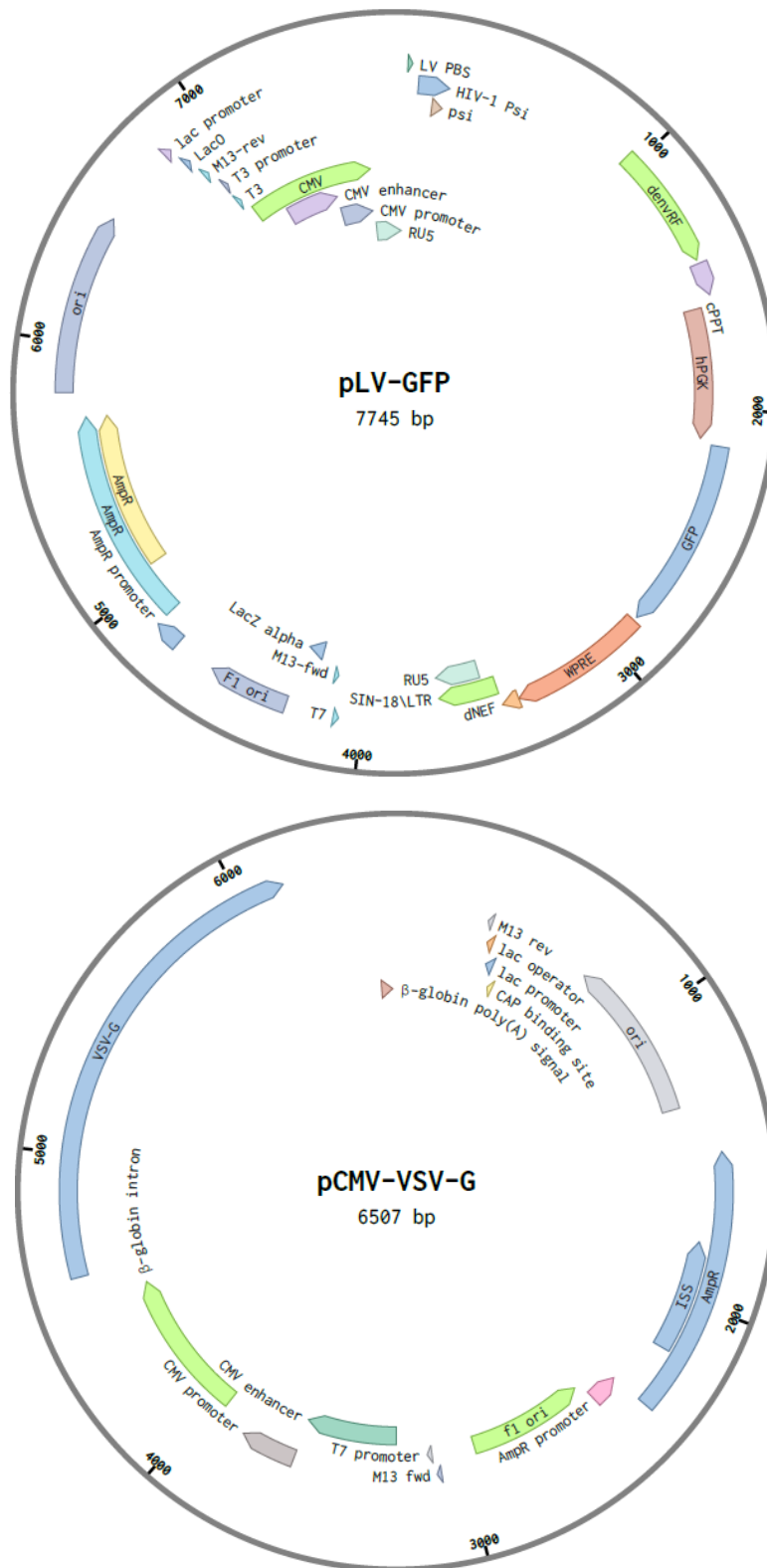

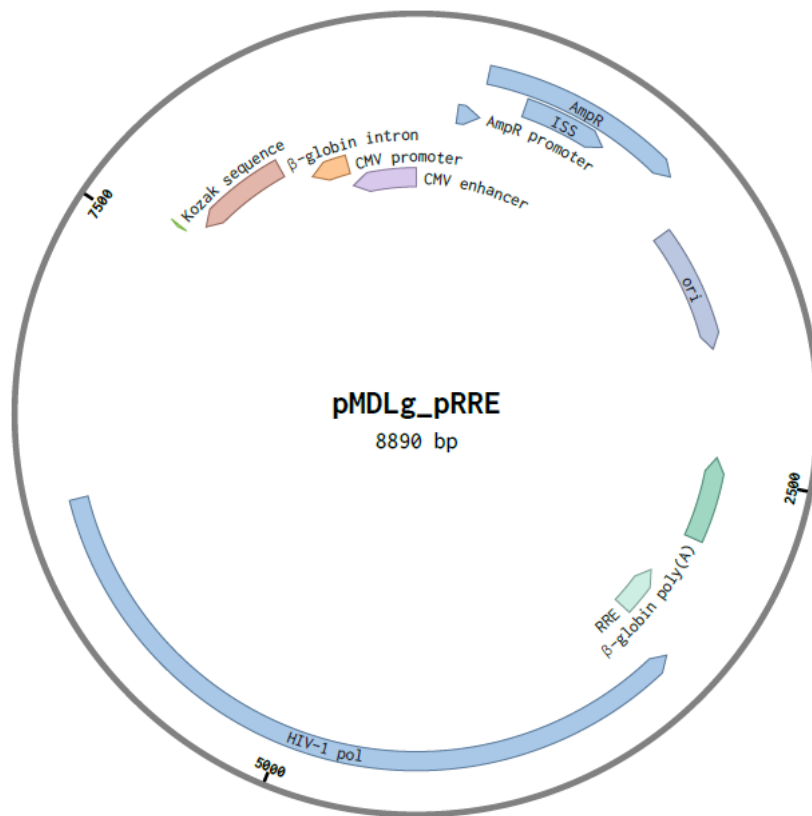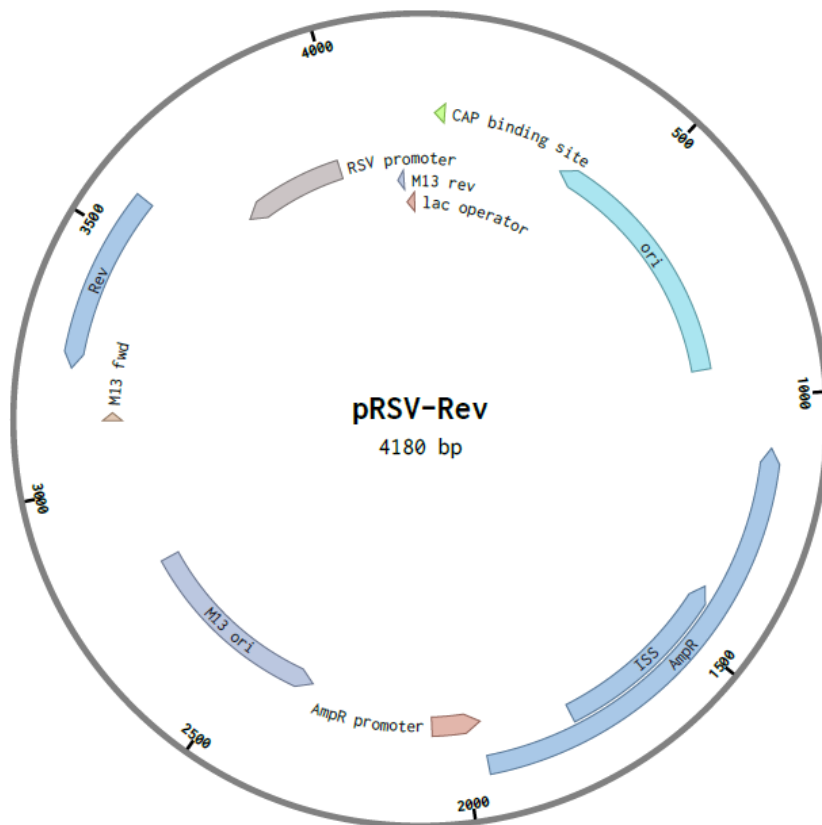

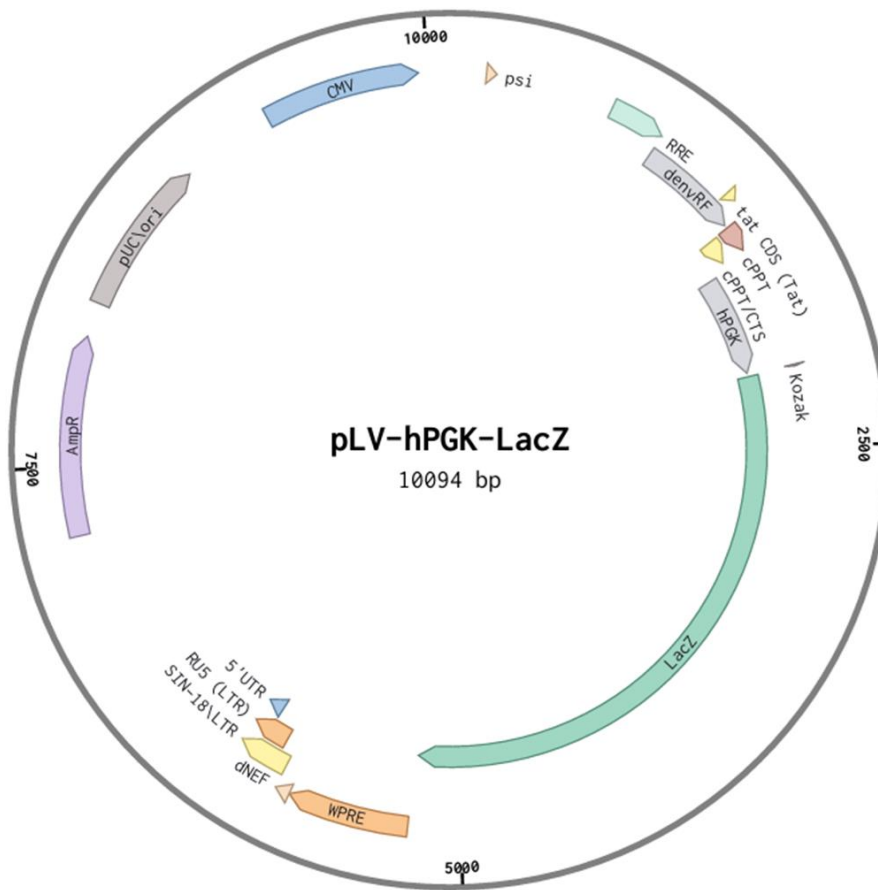

Supplement: Supplementary file 1 [file genes-17-00060-s001.zip › Supplementary File S1.pdf]
